# Balancing bulk gas accumulation and gas output before and during lava fountaining episodes at Mt. Etna

**DOI:** 10.1038/srep18049

**Published:** 2015-12-10

**Authors:** Daniele Carbone, Luciano Zuccarello, Alfio Messina, Simona Scollo, Hazel Rymer

**Affiliations:** 1 Istituto Nazionale di Geofisica e Vulcanologia, Sezione di Catania - Osservatorio Etneo, Catania, Italy; 2 Isituto Nazionale di Geofisica e Vulcanologia, Sezione di Roma 2, Roma, Italy; 3 The Open University, Milton Keynes, UK

## Abstract

We focus on a sequence of 9 lava fountains from Etna that occurred in 2011, separated by intervals of 5 to 10 days. Continuous measurements allowed to discover the occurrence of gravity decreases before the onset of most fountaining episodes. We propose that the gravity changes are due to the pre-fountaining accumulation of a foam layer at shallow levels in the plumbing system of the volcano. Relying on the relationship between amount of gas trapped in the foam and amount of gas emitted during each episode, we develop a conceptual model of the mechanism controlling the passage from Strombolian to lava fountaining activity. Gas leakage from the foam layer during the late stages of its accumulation increases the gas volume fraction at upper levels, thus inducing a decrease of the magma-static pressure in the trapping zone and a further growth of the foam. This feedback mechanism eventually leads to the collapse of the foam layer and to the onset of lava fountaining. The possibility to detect the development of a foam layer at depth and to set quantitative constraints on the amount of trapped gas is important because of the implications for forecasting explosive eruptions and predicting their intensity.

Mt Etna, one the most active volcanoes in Europe, is located on the east coast of Sicily (Italy). Its recent volcanic activity has been mainly focused on the summit craters (Fig. 1) and along fissures on the flanks^1^,^2^. Lava fountains have been one of the most distinctive characters of Etna’s volcanic activity during the last decade^3^,^4^. Since January 2011 and up to the date of this writing, 45 episodes occurred from the New Southeast Crater^5^ (NSEC; Fig. 1), that developed on the flank of the older Southeast Crater (SEC; Fig. 1) and, since 2009, became the new focus of the fountaining activity. Etna’s lava fountains are especially relevant because of the large amount of ash fallout they produce, that represents a major threat both to infrastructures in the villages and towns around the volcano^6^,^7^,^8^ and to aviation. Indeed, during several fountaining episodes, the presence of the volcanic plume in the atmosphere forced the closure of the International Airport of Catania^9^.

The present study is focused on the sequence of paroxysmal eruptive episodes that occurred during the summer of 2011, when the intervals between successive events, usually of the order of few weeks to several months, dropped to only 5–10 days^5^,^10^ (Fig. 2). The summer 2011 fountaining episodes were all confined to the NSEC (and vents on its flanks) and, like other previous and subsequent similar events, displayed a consistent pattern of activity, including, a reactivation phase, with explosive activity at low levels, followed by Strombolian activity, lasting a few hours to several days and leading, through a regular increase in the frequency and intensity of the explosions, to the onset of the fountaining activity.

Mt Etna is one of the most closely monitored volcanoes in the world. Several parameters are permanently measured by the Istituto Nazionale di Geofisica e Vulcanologia - Osservatorio Etneo, Sezione di Catania (INGV-OE; www.ct.ingv.it), that also operates visual and thermal cameras on the flanks of the volcano. The latter are widely used to characterize explosive and effusive events and quantify some eruptive parameters, like the onset time of the paroxysmal episodes and the evolving geometrical parameters of the eruptive columns and lava flows^3^,^4^. Mt Etna is also one of only a few volcanoes in the world where continuous gravity measurements are routinely performed^11^,^12^,^13^.

In this study we shed new light on the mechanisms that drive fountaining activity at Mt. Etna by crossing information from gravity data and thermal imagery. We use the data from a continuous gravity station very close to the summit craters to retrieve a first-order estimate of the bulk gas volume that accumulates in the shallow reservoir prior to each lava fountain. We also estimate the average volume of gas expelled during each fountaining episode. Finally, we develop a conceptual model of the mechanism controlling the passage from Strombolian to fountaining activity and discuss the implication of our results with respect to the possibility of forecasting the occurrence and intensity of explosive eruptions.

## Results

### Gravity data

**Figure 1:**
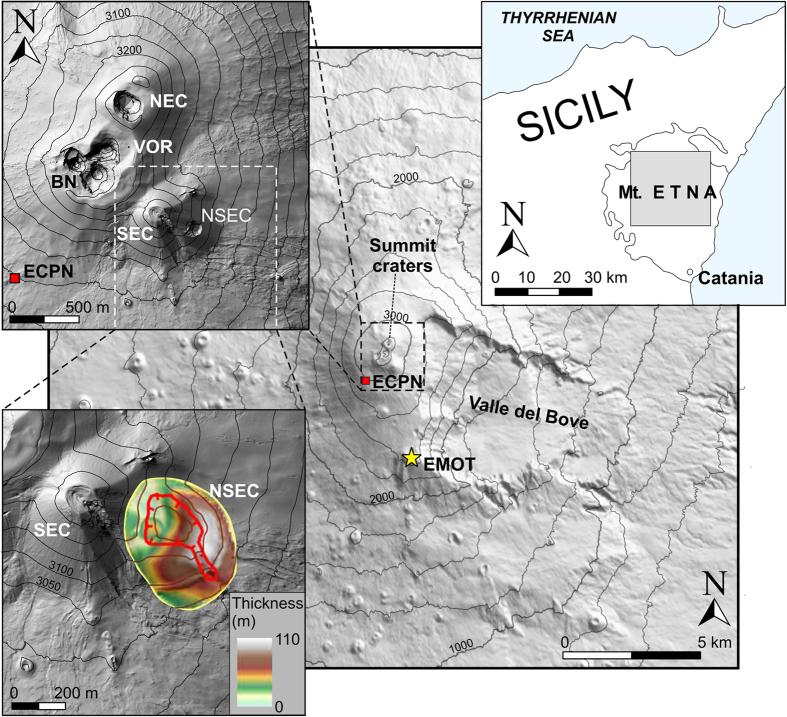
Map of Mount Etna. Yellow star: thermal and visible cameras at La Montagnola (EMOT); red square: continuously recording gravity station (ECPN). The inset at the top right shows the position of Mt. Etna in the eastern part of Sicily (Italy; the gray square indicates the area enclosed by the central panel of the figure). The inset at the top left shows a detail of the craters in the summit zone of Mt Etna (NEC, Northeastern Crater; VOR, Voragine; BN, Bocca Nuova; SEC, Southeastern Crater; NCES New Southeastern Crater). Note that, during the early stages of its development (since November 2009), the NSEC was a pit crater. Lava fountaining activity since January 2011 led to the construction of a pyroclastic cone. In particular, the inset at the bottom left shows the morphological reconstruction of the NSEC scoria cone at the end of the period under study (September 2011; redrawn after Behncke *et al*., 2014). The maps in the figure were generated through the *Surfer*® software (version 8), using a DEM owned by INGV.

On the 1^st^ of July 2011, a LaCoste and Romberg spring gravimeter (model: D; ser. num.: 162) was installed in the summit zone of Etna, where it worked continuously until 12 September. The sensor was installed only ~1 km away from the active summit craters, in a site (ECPN station; Fig. 1) where other instruments were already recording (a broad-band seismometer and a GPS receiver); these instruments are part of the permanent monitoring network operated by INGV-OE. Gravity data were acquired at a rate of 1 Hz. Data are corrected for the effect of Earth tides, through the Eterna33 software package^14^. The effect of instrumental drift is reduced using a polynomial filter that removes the lower-frequency components of the signal (T > ~15 days). Finally, residual artifacts driven by ambient temperature are compensated through the method described in Andò and Carbone^15^.

**Figure 2:**
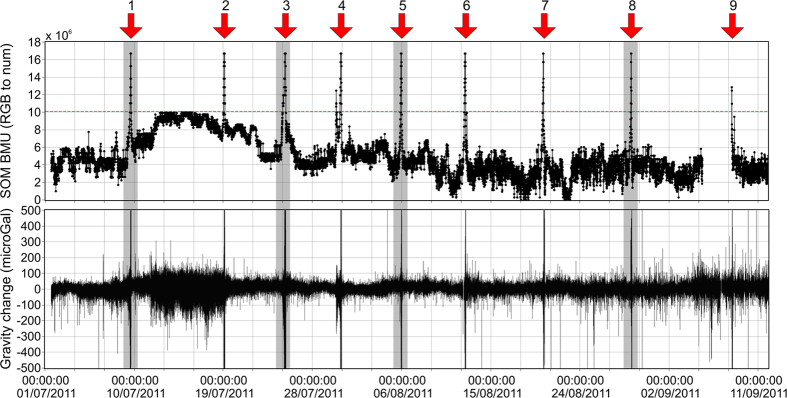
Bottom: gravity signal observed at ECPN station (see Fig. 1), between 1 July and 12 September 2011. The signal is corrected for effect of Earth tides, instrumental drift and residual artifacts driven by ambient temperature (see text for details). Top: SOM *best matching units* (BMUs) for each 5-min interval, after conversion of the RGB colors to numbers (see text for details). BMUs above the dashed red line represent the subset of patterns that are indicative of critical “*ground shaking conditions*” (see text and Fig. 5). Red arrows point to lava fountaining episodes. Grey strips indicate the parts of the signal reported in the panels of Fig. 3.

The gravity signal (Fig. 2) encompasses 9 episodes of lava fountaining (see Fig. 2 and Table 1). Overall, the amplitude of the higher-frequency component (cut off equal to 0.01 Hz) of the signal is equal to about 25 μGal. This value increases to about 80 μGal during the 11–19 July interval, when Strombolian activity occurred from BN crater (Fig. 1), and up to some hundreds of μGal during the lava fountaining episodes (Fig. 2). In the present study we focus our attention on the gravity changes that take place some hours before each lava fountain episode and we disregard variations over longer periods. Indeed, we are interested in the gravity effect of the shallow processes that are directly involved in the onset of lava fountaining activity.

**Figure 3:**
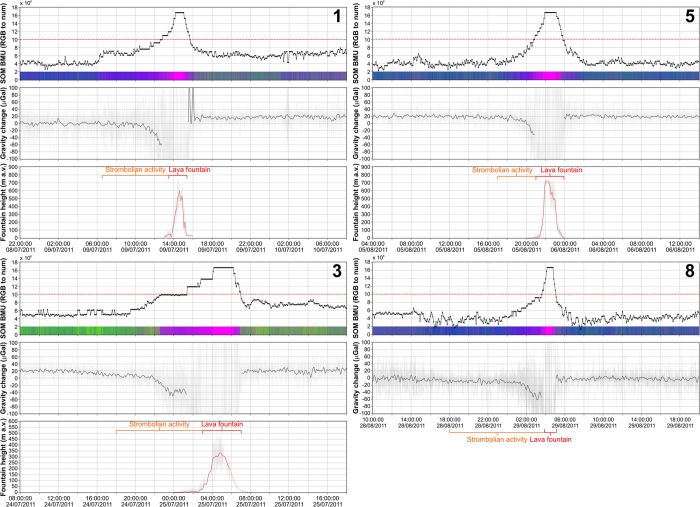
SOM *best matching units* for the classified patterns (RGB colors converted to integer values; see Method section), gravity and fountain height (meters above the vent) during four 34-hour intervals encompassing lava fountaining episodes (numbers correspond to the sequential numbering in Fig. 2). The dashed red lines and color bars in the top graphs of each panel represent, respectively, (i) the threshold above which the SOM patterns are indicative of critical “*ground shaking conditions*” and gravity data are disregarded (lacking filtered data in the gravity graphs) and (ii) the RGB colors of the SOM best matching units for the patterns classified during the four time intervals (see Method section). Intervals when Strombolian and fountaining activity took place are reported in the lower graphs of each panel. The height of the 29 August fountaining episode could not be calculated through our automated procedure since it occurred from an extended fissure along the flank of the NSEC, rather than from a single vent (see text for details).

Unfortunately, during the paroxysmal stages of the lava fountaining activity, the severe ground shaking produced in the near-field by the interactions between magmatic fluids and surrounding rocks^16^,^17^ corrupts the gravity signal to the extent of unintelligibility. Indeed, because of resonance effects^18^, the amplitude of the higher frequency component of the signal (periods ranging from the sampling interval to several minutes) increases to more than 10 times higher than the amplitude of the expected gravity changes. We are thus forced to exclude from our analysis data collected during the phases when the strongest ground shaking is produced. In order to establish the threshold over which data are neglected, we use the seismic signal from a broadband station co-located with (i.e., within 2 m from) the gravimeter. As shown in previous papers^19^,^20^, the effect of horizontal and vertical ground motions on the output from a continuously recording gravimeter depends on the amplitude and spectral content of the exciting seismic signal. To take into account as much as possible information on the disturbing ground shaking and to ensure that the above threshold is objectively valid, we utilize the results of an unsupervised pattern recognition scheme. In particular, we use the KKAnalysis software^21^ to recognize patterns with comparable characteristics, i.e., “*ground shaking conditions*” very similar to each-other (see Method section). Eventually, by cross-checking gravity data with results from KKAnalysis, we identify the patterns that are indicative of critical “*ground shaking conditions*” and we exclude from further analyses gravity data acquired during intervals when such conditions are met (Figs 2 and 3).

In general, a gravity decrease of between 45 and 65 μGal occurs during the last few hours before the onset of each lava fountain (Fig. 3 and Table 1), i.e., during the phases of Strombolian activity preceding the paroxysmal episodes^5^. This pattern is clearly recognizable before most lava fountaining episodes (Fig. 3). Exceptions occur for the 19 July and 30 July episodes. In both cases, gravity changes before the paroxysmal phases of the activity cannot be reliably observed because of unfavorable signal-to-noise ratios.

The observed gravity changes are negligibly affected by the ensuing ground deformation. Indeed analysis of the data from the GPS station in the same site as the gravimeter (ECPN; Fig. 1) and from the electronic levels fitted in the gravimeter itself (resolution = 2.5 μrad^12^) reveals that, before and during the lava fountaining episodes, elevation and tilt changes remained within a few cm and a few tens of μrad, respectively, implying a gravity effect within a few μGal. Aiuppa *et al*.^22^ also found small ground deformation (less than 1 cm) associated with the 2008 lava fountains of Etna.

The need to exclude from our analysis gravity data collected during the paroxysmal phases of the activity implies a higher degree of uncertainty on the average amplitude of the observed gravity decreases. Indeed, it is not possible to tell how each anomaly would have evolved during and after the onset of the fountaining activity. Further discussion on this issue is given below.

### Amount of gas accumulated at depth, deduced from gravity data

Previous studies based on geophysical, geochemical and volcanological observations^5^,^10^,^23^,^24^,^25^ concluded that the episodes of lava fountaining at Etna are triggered by massive collapses of a foam layer that accumulates at shallow depth. Behncke *et al*.^5^ suggested that the foam layer is rebuilt prior to each fountaining episode^26^, in agreement with the collapsing foam model of Jaupart and Vergniolle^27^.

We speculate that the phases of growth of the foam layer caused the gravity decreases observed before most lava fountains during the studied period. Indeed, as already shown by Carbone *et al*.^12^, when gas bubbles substitute a denser material (magma), a localized mass decrease occurs, which is detectable at the surface as a gravity decrease.

As noted before, the average amplitude of the observed gravity decreases could be affected by the lack of data during the phases of strongest activity. Behncke *et al*.^5^ described the onset of most episodes of the 2011 Etna’s fountaining activity as a gradual increase in the frequency and intensity of Strombolian explosions, eventually blending into a continuous jet. Hence, it is reasonable to envisage, before each fountaining episode, a process where the dynamic balance between foam growth and gas flow towards the NSEC conduit is progressively altered in favor of the latter, until the volume of the foam is prevented from increasing more. The hypothesis that the observed gravity decreases reflect the entire process of gas-to-magma substitution before each fountaining episode implies that the volume of the foam does not increase (or increases negligibly) during the intervals when gravity data are excluded due to contamination from ground shaking. The validity of this assumption is supported by the observation that the strong ground motion leading to the exclusion of contaminated data segments is likely driven by sustained gas flux from the trapping zone to the atmosphere through the conduit system of the NSEC.

Under the above hypothesis about the process behind the observed gravity decreases, it is possible to roughly estimate the amount of gas trapped in the foam using the average amplitude of the gravity changes. This calculation requires assumptions about the position and shape of the gravity source and also about the density contrast between substituting and substituted material. Constraints on the position of the gravity source cannot be set using data from only one station. However, following the hypothesis about the pre-fountaining gravity decreases being driven by the accumulation of a foam layer, we can exploit the available independent information on the position of the trapping zone in the shallow part of Etna’s plumbing system. Most of this information comes from geophysical^10^,^28^,^29^ and geochemical^22^,^23^ observations. Based on the relatively high CO_2_/SO_2_ ratios of intra-eruptive, quiescent gas emissions from the summit craters of Etna, Aiuppa *et al*.^22^ suggested that bubbles accumulation occurs at very shallow depth, i.e., at around 2 km asl. This roughly agrees with previous findings^23^ based on the FTIR-sensed composition of lava fountaining gas jets. Tremor source locations, obtained by inverting the spatial distribution of volcanic tremor amplitudes, suggest that the shallow magma storage zone feeding the fountaining activity from the NSEC is not located below the same crater, but, rather, to the NW of it^10^,^22^,^28^, i.e., below the area occupied by the central craters (BN and VOR; Fig. 1) complex (thereafter, CCs complex). As noted by Aiuppa *et al*.^22^ and by Patanè *et al*.^10^, LP and VLP events are located above this zone and could result from gas bubbles that are released from the shallow magma storage and feed surface gas emission^30^. Once the bubble layer collapses, the foamy magma rapidly ascents towards SE, from the trapping zone below the CCs complex towards the NSEC, eventually triggering the fountaining activity. This magma transfer occurs in the framework of the branched structure of the shallow plumbing system of Etna^29^, whose development is thought to be controlled by the local stress field in the summit zone of the volcano^22^,^31^. Indeed, some evidences suggest that the NW-SE-trending fracture systems on the volcano’s summit plays an important role in the movements of magma at shallow levels. To constrain the mass change needed to induce the observed pre-fountaining gravity decreases, we assume a spherically-shaped gravity source placed below the CCs complex area, at a depth of about 1200 m below the ground surface (~2 km a.s.l.). The foam accumulation must cause a local mass decrease of between 2.0 and 3.2 × 10^10^ kg to induce a ~50 μGal decrease at the observation point. For reasonable values of the local density decrease resulting from gas bubbles-to-magma substitution (2500–2700 kg m^−3^), we obtain a bulk volume of exsolved gas in the foam layer of 8 to 12 × 10^6^ m^3^. This figure is not significantly affected by the shape of the source. Indeed, the assumed density change implies a source size of the order of 100 m, i.e., more than 10 times smaller than the source-to-sensor distance. For example, we calculate that, if the source is assumed to have an oblate spheroidal shape (long-short-axes ratio ≈ 2), for the same position and mass change, the resulting gravity decrease at the observation point is altered by only 1%.

### Amount of gas emitted during lava fountains

The volume of gas (*V*) expelled through a fire fountain can be estimated from the average height (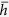) of the fountain, its duration (*t*) and the area of the vent (*s*)^12^,^32^,^33^:


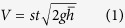


The square root term in (1) is the gas velocity at the vent and results from a balance between kinetic and potential energy^33^.

In order to estimate the gas volume as precisely as possible through (1), instead of considering the average value of the fountain height 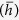, we evaluate its changes over time. As detailed in the Method section, this task is accomplished by analyzing the video streams from the INGV-OE thermal camera located at La Montagnola^10^,^24^ (~3 km South-East of the NSEC, 2610 m asl; EMOT in Fig. 1). The resulting changes in fountain height (at 1 Hz) are low-pass filtered (cut-off frequency ≈ 1 mHz) to reduce the noise (see Fig. 3). The quantity 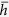 in equation 1 is eventually obtained by integrating the filtered signal over the time period when the fountain takes place:


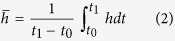


where t_0_ and t_1_ are times of start and end of the lava fountain, respectively (Table 1).

As reported by Behncke *et al*.^5^, the five events between 9 July and 5 August occurred from a vent (or a cluster of vents) within the NSEC, while, since the 12 August episode, active vents opened on the southeastern flank of the growing cone, due to structural weakening, and some of the following events consisted in a curtain of fire from a fissure that extended down to the base of the cone. Hence, for events since 12 August, it is not feasible to calculate the volume of the emitted gas through (1), that assumes eruption from a single vent of given size. Using the procedure described above, we evaluate, for the five episodes between 9 July and 5 August, the quantity (equivalent length):


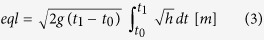


whose value is found to range between 4.5 and 9.4 × 10^5^ m (see Table 1). *eql* represents the time-integration of the exit velocity at the vent. The volume of emitted gas is obtained by multiplying *eql* by *s*.

Calvari *et al*.^24^ reported that, in early 2011, the diameter of the vent at the bottom of the pit crater (the cone of the NSEC had not yet formed at that time) was about 30 m. A larger vent size is deduced from the maps provided in Behncke *et al*.^5^, that showed the evolution of the NSEC between 2009 and 2012. The area of the vent can be deduced from inversion of the mass eruption rate (MER) of a single eruption. We focus on the 9 July episode that, thanks to favorable weather conditions, could be clearly observed by the visible cameras of  INGV-OE surveillance system^9^. During the climactic phase (13:30–16:00, all times are GMT; see Fig. 3), the eruption column reached an height of about 9 km above the vent (estimated by analysis of the images from the calibrated visible camera located in Catania)^4^. This figure can be used to evaluate the MER of the eruption through the model of Degruyter and Bonadonna^34^. Using meteorological data provided by the Italian Air Force Meteorological Office and Civil Protection, a MER of the order of 10^6^ kg s^−1^ is obtained. This value can be used to retrieve the vent size using the equation proposed by Ripepe *et al*.^35^:


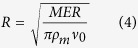


where *R* is the radius of the vent (m), ν_0_ is the plume exit velocity (m s^−1^) and *ρ*_*m*_ is the density of the magma/gas mixture (kg m^−3^). The latter is given by:


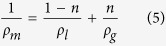


where *ρ*_*l*_ and *ρ*_*g*_ are densities of liquid and gas phases, respectively, while *n* is the volatile content. The mean gas velocity at the vent, ν_0_, can be obtained as 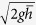
(see above). The average fountain height during the paroxysmal phase of the eruption is 540 m (Fig. 3), giving an exit velocity of  ~100 m/s. Assuming densities of 2700 and 0.15 kg/m^3^ for magma and gas, respectively, and a gas content of 3.4 wt%^36^, a vent radius of 27 m is obtained through (4). Using this value and the above-derived range for the other variables in (1), for each of the five fountains between 9 July and 5 August, we obtain a volume of emitted gas that ranges between 0.9 and 1.7 × 10^9^ m^3^.

## Discussion

The results presented in the previous section about the gas volumes accumulated at depth and released during the fountaining activity can be used to set new constraints on the volcanic processes that regulate Etna’s lava fountaining activity.

If dynamic terms^37^,^38^ are neglected for the sake of simplicity, the pressure at the shallow reservoir where the foam layer accumulates can be calculated from the magma-static equation^39^,^40^:


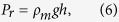


where *h* is the depth of the reservoir (m) and *g* the acceleration of gravity (m s^−2^). Equation (6) can be rewritten as:


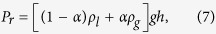


where α is the gas volume fraction. Since *ρ*_*g*_is much lower than *ρ*_*l*_, we have^41^:


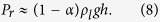


**Figure 4:**
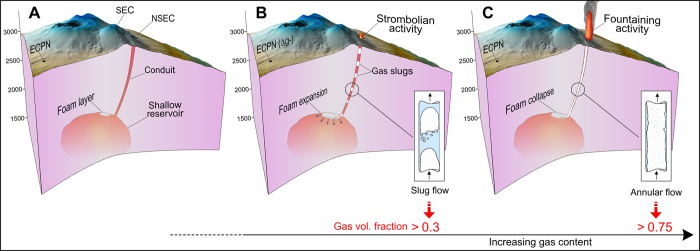
(**A**) The first stages of accumulation of the foam layer in the shallow reservoir take place during phases of quiescent degassing from the summit craters. (**B**) At later stages of the foam accumulation, gas leakage occurs from the foam and bubbles flow towards the conduit, forming gas pockets at the conduit entrance. The flow regime gradually changes from bubbly to slug flow, leading to the onset of Strombolian activity. (**C**) The feed-back loop involving the increase of the gas volume fraction in the conduit and the decrease of *P*_*r*_, results in further growth of the foam that eventually collapses. Transition from slug to annular flow takes place in the conduit marking the passage from Strombolian to lava fountaining activity. Increasingly higher seismic energy is radiated and the signal from the gravimeter is severely degraded by inertial effects. The position of the gravity station (ECPN) in the three panels is marked by the black dot. SEC and NSEC indicate Southeastern and New Southeastern Crater, respectively (see Fig. 1). The maps in the figure were generated through the *Surfer*® software (version 8), using a DEM owned by INGV.

As reported in the previous section, the eruptive episodes during the summer of 2011 showed a similar succession of different phases, including reactivation with minor explosive activity and, subsequently, Strombolian activity, with a gradual increase in the frequency and intensity of the explosions, until the onset of the fountaining activity. Lava emission typically preceded the onset of lava fountaining by a few tens of minutes to several hours^5^. Models of separated two-phase flow through a conduit^42^ predict that these different stages of activity correspond to different patterns of gas-liquid flow. In particular, the onset of Strombolian activity marks the passage from the bubbly to the slug regime, while fountaining activity occurs at the transition from the slug to the annular pattern^43^ (Fig. 4). The different flow regimes and the transitions between them depend on many parameters, one of the most important being the volume fraction of the gas in the conduit^41^,^44^. In particular, slug flow (hence, Strombolian activity) cannot develop below gas volume fractions of about 0.3, while annular flow (fountaining activity) requires values higher than about 0.7^45^ (Fig. 4).

As stated before, previous studies concluded that the episodes of lava fountaining at Etna are produced by the violent eruption of a gas bubble layer previously accumulated at shallow depth^5^,^10^,^12^,^23^,^24^,^25^,^26^,^27^. We thus hypothesize that the amount of gas emitted during each fountaining episode (*V*_1_; see *Results*) corresponds to the amount of gas previously accumulated in the foam layer at depth (*V*_2_; see *Results*). Assuming ideal gas behavior and isothermal conditions, we can thus roughly estimate the pressure at the shallow reservoir as:


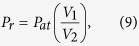


where *P*_*at*_ is atmospheric pressure. Using the values for *V*_1_ and *V*_2_ reported above, a value of 14 ± 7 MPa for *P*_*r*_ is obtained, which, according to equation (8) and assuming *h* ≈ 1200 m and *ρ*_*l*_ = 2700 kg m^−3^, corresponds to a gas volume fraction (α) of 0.5 ± 0.2. In the above calculations we do not take into account the gas still dissolved in the melt when the switch from Strombolian to fountaining activity occurs. If this gas then exsolves from the melt and is emitted during the fountaining activity, we need not to consider it in equation (9), implying that we must subtract it from *V*_*1*_. For an initial water content of 3.4 wt%^36^, about 20% of water is still dissolved in the melt at a pressure of about 14 MPa^46^. If *V*_*1*_ is lowered by 20% (from 0.9–1.7 to 0.7–1.4 * 10^9^ m^3^), the average value of *P*_*r*_ decreases by 2 to 3 MPa and α increases by about 0.1 (from 0.5 to 0.6 ± 0.2).

Within the limits of the assumptions made, from the above result the following conclusions can be drawn. A feedback relationship exists between foam growth and transition between the different regimes, until the onset of the fountaining activity. The first stages of accumulation of the foam layer are likely to take place during phases of quiescent degassing from the summit craters (Fig. 4). Under these conditions, no pressure decrease is likely to occur in the shallow reservoir^39^. Analog experiments^47^ showed that, at late stages of the foam accumulation, gas leakage occurs from the foam itself, with bubbles flowing towards the conduit and coalescing into gas pockets at the conduit entrance. The continuation of this process results in the transition from the bubbly to the slug regime. The above cited feedback loop then starts: the increasing gas fraction in the conduit induces a pressure decrease in the shallow reservoir that, in turn, induces further growth of the foam through both diffusion and decompression-driven expansion of the gas bubbles. The mass decrease in the shallow reservoir induces a gravity change that becomes more and more important as the volume ratio between exsolved gas and magma increases, thus, as *P*_*r*_ decreases. That explains why the pre-fountaining gravity decreases reach the maximum amplitude during phases of Strombolian activity (Fig. 3). As the flow pattern in the conduit evolves from the slug to the annular regime, increasingly larger amounts of seismic energy is radiated^10^,^22^, until a level where the disturbance due to inertial effects prevents further exploitation of the gravity signal. In agreement with this view, the gas volume fraction deduced from the inversion of the available data (α ≈ 0.5 ÷ 0.6) suggests that most of the pre-fountaining gravity decrease develops when the flow pattern in the conduit is somewhere between the bubbly/slug and the slug/annular transitions (Fig. 4).

As noted in previous studies^12^,^13^,^48^, we show that continuous gravity observations can be used to detect fast changes in the relative proportions of magma and exsolved gas in the shallow levels of the plumbing system. They are thus important both to early recognize phases of gas accumulation that may lead to energetic eruptive episodes and also to set quantitative constraints on the amount of exsolved gas trapped at depth. The latter information is especially important being directly related to the strength and dangerousness of the impending explosive eruption. In order to avoid the shortcomings induced by severe ground shaking in the near-field, the possibilities of vibration isolation systems to improve the signal from continuously running spring gravimeters could be tested. Alternatively, superconducting gravimetrs could be employed. Since they feature a much higher stability and precision than provided by spring-type meters^49^, they could detect volcano-related gravity changes even at sites far from the active craters, where a reliable (gravity) signal - to - (seismic) noise ratio could be obtained, even during paroxysmal phases of the activity.

## Methods

### *Ground shaking conditions* defined through the KKAnalysis software

To establish the threshold over which gravity data are neglected due to the disturbance from ground shaking, we use the KKAnalysis software^21^, which exploits the Self-Organizing Map (SOM)^50^ method to classify datasets of multidimensional patterns (feature vectors). We use the seismic signal (sampled at 100 Hz) from a broadband station co-located with the gravimeter. Before running the program, we convert the seismic signal into a discrete sequence of more than 2 × 10^4^ patterns, through the following steps:

1. in a 1024-sample window, sliding along the seismic signal with 500-sample overlap, discrete spectral amplitudes are calculated in frequency bins of 0.29 Hz;
2. a series of 60 vectors (one every 5 seconds) of spectral amplitudes is obtained for each 5-minute interval;
3. each series is converted into a single pattern by calculating the 10^th^ percentile of the spectral distribution.

The above steps are repeated for each of the three components (two horizontal, one vertical) of the seismic signal. For each 5-minute interval, the three resulting patterns (one for each component) are eventually merged into a single pattern. The latter represents the “*ground shaking conditions*” for a given 5-minute interval of the time sequence, deduced on the ground of amplitude and spectral content of the seismic signal.

**Figure 5:**
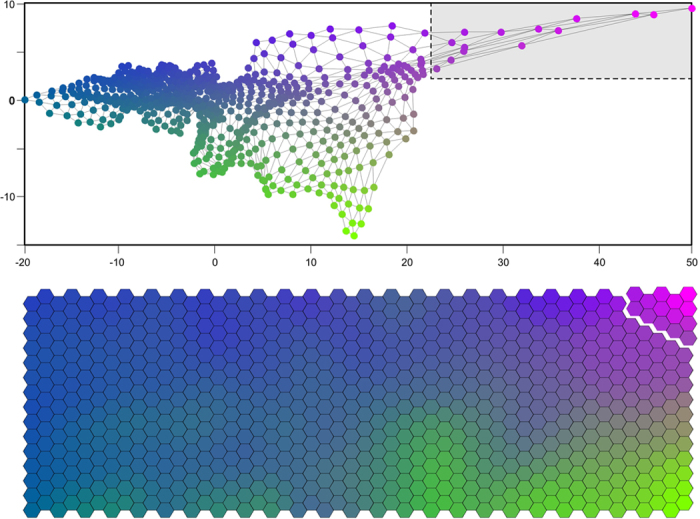
SOM representations. Top: 2D projection of weights of the SOM in a system of axes spanned by the two major principal components (formally dimensionless). Bottom: 2D map of our 800 SOM nodes, representing neighborhood relations schematically. Each hexagon represents the *best matching unit* (BMU) for a given number of patterns (see text for details). BMUs inside the dashed rectangle (top) and detached from the map (bottom) represent the subset of patterns that are indicative of critical “*ground shaking conditions*”.

The set of generated patterns forms the input to KKAnalysis. The program creates a SOM of 15 × 50 nodes of the same dimensionality as the input patterns (Fig. 5). The SOM is generated by an iterative scheme aimed at identifying the best matching unit (BMU) for each pattern. The BMU represents the closest SOM node to the actual feature vector^21^. During the iterative process, the node weights are gradually adjusted until a stable configuration of the SOM is obtained. Interestingly, the topological relationship of the original data space is maintained in the SOM; indeed, patterns represented by neighboring nodes in the SOM are also close to each other in the original data space. A RGB color is then assigned to each node of the SOM, through preforming a principal component analysis (PCA). Through the pattern classification performed by KKAnalysis it is therefore possible to recognize patterns with comparable characteristics (that are represented by the same node/color, or by neighbor nodes/colors, in the SOM), i.e., “*ground shaking conditions*” very similar to each-other. By cross-checking gravity data with results from KKAnalysis, we determine which nodes of the output SOM correspond to corrupted portions of the gravity signal (Figs 3 and 5). In other words, we identify the subset of SOM nodes (i.e., the subset of patterns) that are indicative of critical “*ground shaking conditions*”.

### Lava fountain height evaluated from thermal imagery

**Figure 6:**
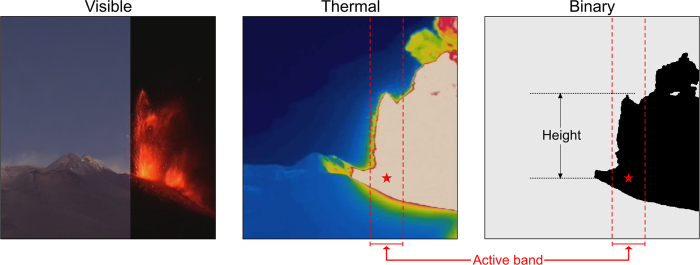
Left: composed visible image from the camera at La Montagnola (EMOT; Fig. 1) with a part of the image acquired under daylight conditions (left side) and a part of the frame taken at 19:57:46 of 30 July 2011 (right side). Middle: corresponding part of the thermal image taken at the same site at 19:57:46 of 30 July 2011. Right: binary image obtained from the corresponding thermal image as described in the text. The dashed red lines mark the position of the active band within which the fountain height is determined. The red star marks the position of the active vent (NCSE).

The 320 × 240 pixel images (1 Hz rate) from the EMOT thermal camera (Fig. 1) feature a fixed color scale that ranges between −20 and 60 °C^24^. Under the assumption that during a lava fountain the saturated region (displayed in white; see middle panel in Fig. 6) of the thermal images represents the sustained jets of liquid magma and gas, to retrieve information on the fountain shape, we determine which pixels fall in that region. The latter task is carried out through a C++ code that exploits the OpenCV library (http://opencv.org) for image processing. The procedure involves 2 steps: (1) each frame is converted from the RGB to the HSV standard; (2) the HSV images are converted into binary matrices using the *thresholding* function of OpenCV. By choosing suitable threshold values, we obtain binary images where pixels in the saturated region are set to 1, while all the other pixels are set to 0 (right panel in Fig. 6). For each frame, the fountain height is taken as the height of the region of 1-valued pixels above the vent (Fig. 6). In order to exclude unwanted bordering information, only a narrow vertical band (active band; width = 70 pixels; Fig. 6), centered around the vent position, is considered when determining the fountain height. This choice also permits to distinguish the momentum-driven jets from the buoyant region of the eruption column, which is often pushed by the wind outside the active vertical band (see Fig. 6) and thus not considered in the determination of the fountain height.

Using reference points in the images whose positions are known, a scale factor is deduced allowing to convert the resulting values from pixels to meters.

It is important to stress that the fallout of cooled ash and/or the passage of clouds can hide the top of the lava fountain, thus biasing the results from the above-described procedure. Further uncertainties may arise from (i) the thresholds chosen to convert HSV into binary images, (ii) the perspective and lens distortions and (iii) the presence of buoyancy-driven hot gas falling within the active band.

## Additional Information

**How to cite this article**: Carbone, D. *et al*. Balancing bulk gas accumulation and gas output before and during lava fountaining episodes at Mt. Etna. *Sci. Rep*. **5**, 18049; doi: 10.1038/srep18049 (2015).

## Figures and Tables

**Table 1:** Lava fountain episodes from the NCSE during the studied period.

	Date	Max Ampl. Δg [μGal]	*V*_*2*_ [10^6^ m^3^]	*eql* [m]	Start Strombolian	Start time of no gravity data	Start Fountaining	End Fountaining
1	09/07/11	65	13	4.5 × 10^5^	07/07/11 20:00	09/07/11 12:45	09/07/11 14:00	09/07/11 15:15
2	19/07/11	—	—	5.7 × 10^5^	18/07/11 17:00	—	19/07/11 00:05	19/07/11 02:30
3	25/07/11	60	12	9.4 × 10^5^	24/07/11 18:00	25/07/11 01:15	25/07/11 03:00	25/07/11 06:00
4	30/07/11	—	—	7.0 × 10^5^	30/07/11 07:50	—	30/07/11 19:35	30/07/11 21:45
5	05/08/11	50	10	7.3 × 10^5^	05/08/11 17:00	05/08/11 20:50	05/08/11 21:45	05/08/11 23:00
6	12/08/11	55	11	—	12/08/11 05:30	12/08/11 07:30	12/08/11 08:30	12/08/11 10:45
7	20/08/11	45	9	—	20/08/11 02:00	20/08/11 06:00	20/08/11 07:00	20/08/11 07:50
8	29/08/11	50	10	—	28/08/11 18:00	29/08/11 02:45	29/08/11 04:05	29/08/11 04:50
9	08/09/11	60	12	—	08/09/11 05:30	08/09/11 06:40	08/09/11 07:20	08/09/11 08:30

The maximum amplitude of the gravity decreases and the volume of gas accumulated at depth prior to each fountaining episode (*V*_*2*_ in eq. 9) are reported in column 3 and 4, respectively. The quantity *eql* (eq. 3) for the five episodes between 9 July and 5 August is reported in column 5. The other columns report, for each episode, the timing of start/end of the Strombolian and lava fountaining activity and (column 6) the start time of the intervals when gravity data cannot be used any more because of the disturbance from ground shaking. Note that gravity changes before the 19 and 30 July episodes cannot be reliably observed because of unfavorable signal-to-noise ratios. *eql* was not calculated after the 5 August episode because emission did not occur from a single vent (see text for details).
